# The Feeble-Minded in Poor-Law Infirmaries

**Published:** 1912-05-25

**Authors:** 


### THE FEEBLE-MINDED IN POOR-LAW INFIRMARIES.

A deputation from the Leicester Guardians were recently discussing the question of further accommodation for infirmary patients with the Local Government Board. The Guardians suggested that the imbecile, epileptic, and feeble-minded cases might be taken from the infirmary and placed in a separate building. They were advised to delay action because a Bill was about to be introduced on the subject of the feeble-minded. The Clerk to the Guardians was able to refer to a minute that when a previous deputation, about nine or ten years ago, interviewed the Local Government Board concerning accommodation for the feeble-minded, a similar answer was given. On this occasion the Guardians have not had long to wait.

There are at present two Bills before Parliament dealing with the mentally defective. One, entitled the Feeble-minded Persons (Control) Bill, which was presented by Mr. Stewart, has passed the second reading. The other, called the Mental Deficiency Bill, was introduced by Mr. McKenna for the Government. As may be gathered from the titles of the two Bills, the latter is by far the wider and more comprehensive, both as regards the persons to be dealt with and the means for doing so. When we remember that, according to the statement of the National Association for the Feeble-minded, from 10 to 20 per cent. of our prisoners, 62 per cent. of the inhabitants of the inebriate homes, 50 per cent. of the inmates of rescue homes, and 20 per cent. of those in workhouses are mentally defective, the vastness of the question and the importance of dealing with it broadly and adequately are apparent. The Feeble-minded Persons (Control) Bill excludes idiots and imbeciles from its scope. It also requires, before action under it can be taken, that the feeble-minded person must be shown to be a source of injury and mischief to himself or others. One of the most distressing features of the whole question is the amount of illegitimacy among the feeble-minded women in our workhouses. The Commissions of Lunacy, in dealing with this matter, have pointed out that persons of this class residing in workhouses could be, and ought to be, certified for detention there. In several instances their recommendations to this effect received replies that, as the cases to which attention had been called were quiet and harmless, the medical officer considered certification unnecessary. At present the powers of certification given by the Lunacy Acts are not fully taken advantage of as regards weak-minded women and girls, as well as young imbecile men. Among those who come under the provisions of the Government Bill are those "in whose case it is desirable, in the interests of the community, that they should be deprived of the opportunity of procreating children." Unless the words "source of injury and mischief to himself and others" were interpreted in a very wide sense, it would be impossible to certify the cases referred to by the Commissioners in Lunacy under Mr. Stewart's Bill. From the point of view of the Poor-Law administrator, it is essential that there should be full powers to detain this class. The mentally defective produce more children than normal individuals. The average family of the mentally defective is said to average 7.3, as compared with 4.6 in normal families.

The Government Bill throws upon local authorities the responsibility to provide institutions for the mentally defective. It also promises financial assistance towards the provision of the Homes and the cost of maintaining the inmates. This outlay undoubtedly will be a heavy one. The taxpayer must draw what comfort he can from the thought that he is benefiting posterity. The money spent on this object will not be all additional expense. There will be fewer additions required to our asylums and workhouses. The full advantage will only be reaped by our children's children.

